# Downregulation of Fidgetin-Like 2 Increases Microglial Function: The Relationship Between Microtubules, Morphology, and Activity

**DOI:** 10.1007/s12035-024-04404-0

**Published:** 2024-08-19

**Authors:** Austin N. Smith, Alison Gregor, Lisa Baker, David J. Sharp, Kimberly R. Byrnes

**Affiliations:** 1 Neuroscience Program, Uniformed Services University of the Health Sciences, Bethesda, MD USA; 2 Department of Anatomy, Physiology and Genetics, Uniformed Services University of the Health Sciences, Bethesda, MD USA; 3 MicroCures, Inc., Bronx, NY USA; 4 Department of Molecular Pharmacology, Albert Einstein College of Medicine, Bronx, NY USA

**Keywords:** Cytokine secretion, Cytoskeleton, FIGNL2, Microtubule dynamics, Motility, Nanoparticle siRNA, Phagocytosis

## Abstract

The microtubule cytoskeleton regulates microglial morphology, motility, and effector functions. The microtubule-severing enzyme, fidgetin-like 2 (FL2), negatively regulates cell motility and nerve regeneration, making it a promising therapeutic target for central nervous system injury. Microglia perform important functions in response to inflammation and injury, but how FL2 affects microglia is unclear. In this study, we investigated the role of FL2 in microglial morphology and injury responses in vitro. We first determined that the pro-inflammatory stimulus, lipopolysaccharide (LPS), induced a dose- and time-dependent reduction in FL2 expression associated with reduced microglial ramification. We then administered nanoparticle-encapuslated FL2 siRNA to knockdown FL2 and assess microglial functions compared to negative control siRNA and vehicle controls. Time-lapse live-cell microscopy showed that FL2 knockdown increased the velocity of microglial motility. After incubation with fluorescently labeled IgG-opsonized beads, FL2 knockdown increased phagocytosis. Microglia were exposed to low-dose LPS after nanoparticle treatment to model injury-induced cytokine secretion. FL2 knockdown enhanced LPS-induced cytokine secretion of IL-1α, IL-1β, and TNFα. These results identify FL2 as a regulator of microglial morphology and suggest that FL2 can be targeted to increase or accelerate microglial injury responses.

**Graphical Abstract:**

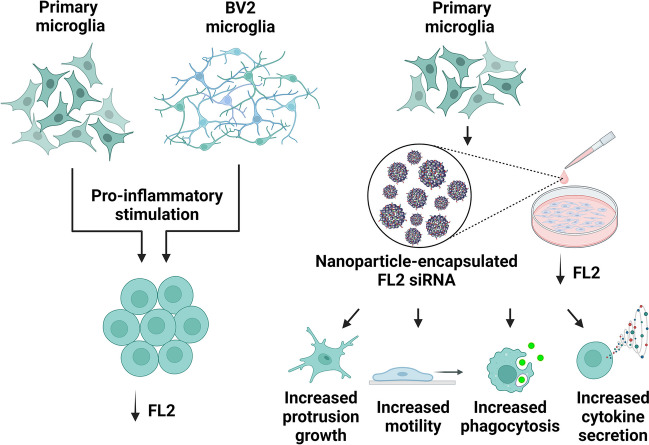

**Supplementary Information:**

The online version contains supplementary material available at 10.1007/s12035-024-04404-0.

## Introduction

Microglia function as resident immune cells of the central nervous system (CNS) [1, 2]. They maintain surveillance of the CNS parenchyma, poised to respond to environmental cues, such as those generated from injury or inflammation. Surveillant microglia exhibit a ramified morphology with the capability of rapidly extending and retracting branched processes [3, 4]. In response to stimuli, microglia become reactive and adopt a more amoeboid form associated with increased motility, phagocytosis, and immunomodulatory release of cytokines and chemokines [5, 6].

The diverse range of microglial morphology and inflammatory phenotypes entails remodeling of the cytoskeleton, composed of microtubules, actin filaments, and vimentin intermediate filaments [7–9]. Major microglial ramifications depend upon microtubule polymerization while narrower filopodia found on the ends of microglial processes are actin-dependent [10, 11]. Microglia upregulate the expression of cytoskeletal proteins and increase the reorganization of cytoskeletal networks in response to various stimuli, such as bacterial lipopolysaccharide (LPS) and inflammatory cytokines [7, 12]. While the contribution of actin dynamics to microglial functions has received substantial attention, further research into microtubule dynamics may provide insight into normal and pathological microglial functions.

Microtubules are αβ-tubulin polymers that regulate microglial function through rapid alternation between phases of growth and shrinkage, a phenomenon known as “dynamic instability” [13]. Ramified microglia bear more stable microtubules but increase microtubule dynamics in response to both pro- and anti-inflammatory cytokines [7, 14–16]. Microtubule polymerization inhibitors impair microglial basal motility [17], chemotaxis [18], branch-mediated endocytosis [17, 19–21], cytokine secretion [16, 22, 23], and suppress the assembly of the NLRP3 inflammasome [24]. Research suggests that increasing microtubule stability also impairs microglial migration, extracellular protein clearance [25], and cytokine secretion [26, 27]. Thus, microtubule dynamics are important to maximize the efficiency of microglial functions.

Fidgetin-like 2 (FL2) belongs to a family of microtubule-severing enzymes that generate internal breaks in microtubules to induce disassembly, repair, or even amplify microtubule numbers to regulate length, branching, and dynamics [28]. FL2 can be found across different tissues among vertebrate species with high expression levels during development found in the CNS [29, 30]. Unlike other fidgetin genes, early FL2 depletion leads to aberrant brain development [31]. Intracellularly, FL2 exhibits increased localization near the leading edge of the cell [32]. Investigations of FL2’s functions found that FL2 severs more dynamic microtubules, suppressing cell growth and motility [32–34]. Administration of FL2 siRNA improved the rate and quality of healing after excision wounds, burns, and chemical injuries [32, 35, 36]. More importantly, the downregulation of FL2 improved functional peripheral nerve regeneration after crush and transection injury [33]. These findings indicate that FL2 is a novel target to regulate CNS cells and treatments targeting FL2 are a potential therapy for CNS injury.

Microglia rely upon microtubule dynamics to establish morphology and respond to stimuli. FL2 regulates microtubule dynamics and is a target to promote wound healing and nerve regeneration. While FL2 provides a novel target for CNS injury, the role of FL2 in CNS cells, including microglia, has not been investigated. In this study, we investigate the role of FL2 expression in microglia. This study is the first to examine FL2 in both a CNS-specific cell and an immune cell.

## Materials and Methods

### Primary Microglia Culture

Mixed glial cultures derived from 2-day-old Sprague–Dawley rats were used to isolate primary microglia as previously described [37]. Cells were cultured in Dulbecco’s Modified Eagle Medium (DMEM; Gibco) supplemented with 10% fetal bovine serum (FBS; R&D Systems) and 1% penicillin–streptomycin (Pen-Strep; Sigma). Media replacement occurred every 3–5 days. Cells were maintained at 37 °C in a humidified incubator with 5% CO_2_. After reaching confluency, mixed glial cultures were shaken in a Benchtop Shaking Incubator (Corning) at 125 rpm for 1 h at 37 °C, yielding high microglial purity. Primary microglia were seeded at 3 × 10^5^ cells/mL density for all experiments and allowed to adhere for 24 h before administering treatments. The purity of microglia isolated by shaking was determined with immunostaining of the microglial marker, CD11b. The proportion of microglia was estimated to be > 95% of cells.

### BV2 Cell Line

The immortalized mouse microglia BV2 cells (a gift from Dr. Carol Colton) were cultured in DMEM with 10% FBS and 1% pen-strep at 37 °C and 5% CO_2_. Cells were passaged with 0.25% Trypsin containing ethylenediaminetetraacetic acid (Sigma) on reaching 80–90% confluence. BV2 cells were seeded at 1 × 10^4^ cells/mL density and allowed to adhere for 24 h before administering treatments. The initial seeding density of BV2 cells was reduced compared to primary microglia to prevent overcrowding due to their high proliferation rate.

### LPS Treatment

LPS from *Escherichia coli* (Sigma) was reconstituted in DI water to a working concentration. Cells were stimulated with a range of LPS (10, 50, or 500 ng/mL) or DI water (control) for 6 h or 24 h.

### Nanoparticle-Encapsulated siRNA Synthesis

The siRNA nanoparticles were synthesized and dosed based on previous in vitro work [33]: 500 μL of tetramethyl orthosilicate (TMOS) was hydrolyzed with 100 μL of 1 mM HCl by sonication on ice for 15 min. TMOS (100 μL) was added to 900 μL of 10 μM of siRNA containing 10 mM phosphate, pH 7.4, and then allowed to solidify into a block gel at room temperature (10–15 min). The metal-oxo skeleton formed by the TMOS entraps the siRNA. The block gel was frozen at − 80 °C, and then lyophilized overnight. The silicate particles were further ground with a mortar and pestle to ensure any larger pieces were further broken up. The lyophilized nanoparticles were then resuspended in PBS and stored at − 80 °C until use. The siRNAs included pooled rat FL2 (siRNA from MilliporeSigma: SASI_Rn02 00314854, target sequence: CTGGATGTCTCCTCCACCA; SASI_Rn02 00314855, target sequence: CAGAGGATGGGACCGGCAA; SASI_Rn02_ 00389576, target sequence: CCTCCAACCTCCTCAAGAG) or the negative control siRNA (MilliporeSigma, Universal Negative Control B). Nanoparticle-encapsulated siRNA in PBS was directly administered to cell cultures at a concentration of 20 nM. FL2 knockdown was validated 24 h after treatment with each batch of nanoparticles. All outcome measures were performed beginning 24 h after siRNA treatments. The nanoparticle-encapsulated FL2 siRNA (SiFi2) and negative control siRNA (SiCon) groups were compared to each other and a PBS (control) group.

### Immunofluorescence Microscopy

Primary microglia were seeded on glass coverslips before treatments and staining. Cells were fixed with 4% paraformaldehyde for 10 min followed by two washes with 1 × PBS. Coverslips were incubated in blocking solution for 15 min and then overnight with primary antibodies against CD11b (1:500, Bio-Rad, MCA275R). The next day, coverslips were washed twice with 1 × PBS and incubated with Alexa Fluor secondary antibodies (1:1000, Invitrogen) for 1 h. Following two washes with 1 × PBS, coverslips were mounted on slides with HardSet Mounting Media with DAPI (Vectashield). Experiments were performed in triplicate and five images (randomly obtained from the center and surrounding four quadrants) were taken on each slide using a fluorescence microscope (Olympus) at 20X magnification.

### RNA Isolation and Gene Expression Analysis

Total RNA was extracted from cells using TRIzol (Invitrogen) and purified using the RNeasy Kit (Qiagen). Complementary DNA (cDNA) was synthesized from RNA with the High-Capacity Reverse Transcriptase Kit (Applied Biosystems) and a Veriti thermal cycler (Applied Biosystems). Reverse transcription quantitative polymerase chain reaction (RT-qPCR) was performed with SYBR Green reagents (Bio-Rad) utilizing a StepOnePlus Real-Time PCR System (Applied Biosciences). Primers were rat and mouse fidgetin-like 2 with phosphoglycerate kinase 1 used as the endogenous reference gene. All primer sequences are listed in Table 1.

**Table 1:** Primers used for RT-qPCR

Gene	Symbol	Species	Sequence 5’-3’	
Fidgetin-like 2	FIGNL2	Rat	Forward Reverse	CTCTGTGCTTCTGTCTCTGT GAGTTGCTGCAGTGTGAATG
Fidgetin-like 2	FIGNL2	Mouse	Forward Reverse	GCTCCTAGATCCCTTCATGTTC GTTCACACTCCTCACACCTG
Phosphoglycerate kinase 1	PGK1	Rat & Mouse	Forward Reverse	GGAGATGTCTATGTCAATGATG TTTAGCTCCTCCCAAGATAG

### Cytotoxicity Assay

The concentration of lactate dehydrogenase (LDH) in the cell culture supernatant was measured as an indicator of cell death using the Cytox 96 Assay (Promega) per manufacturer instructions in triplicate. The absorbance at 492 nm was measured with a ChroMate microplate spectrophotometer (Midwest Scientific).

### Griess Assay

The concentration of nitrite, an end product of nitric oxide metabolism, was measured in the cell culture supernatant to assess nitric oxide production using the Griess Reagent Kit (Invitrogen) per manufacturer instructions in triplicate. The absorbance at 545 nm was measured with a ChroMate microplate spectrophotometer (Midwest Scientific).

### Morphological Analysis

Cells were imaged 24 h after nanoparticle-encapsulated siRNA treatments. Images were taken using a phase-contrast microscope (Zeiss Primovert) with a mounted Swiftcam 5.0-megapixel digital camera microscope (Swift Optical Instruments, Inc.). Five images of each well (randomly obtained from the center and surrounding four quadrants) were taken at 20X magnification. An experimenter who was blinded to treatment groups performed qualitative and quantitative analyses. Microglial morphologies were manually classified into four categories: (1) ramified, (2) hypertrophic, (3) intermediate, or (4) amoeboid. ImageJ was used to quantify cellular morphology characteristics, i.e., cell body area, circularity, number of protrusions, length of protrusions, and number of branching points.

### Live-Cell Imaging

Primary microglia were seeded on plastic bottom 24 well plates. Live-cell imaging was performed beginning at 24 h after nanoparticle-encapsulated siRNA treatment. Images from 4 regions of interest centered around the middle of the well were taken at 10X magnification over 6 h with 10-min intervals using a Cytation 5 incubator microscope (BioTek). The series of time-lapse images were combined into a hyperstack in ImageJ. The hyperstack images were stabilized to correct for drift in the image sequence. 30 random cells were tracked per ROI per treatment group for each experiment. Motility tracks centered within the cell somas were generated using the NIH Manual Tracking script. The Chemotaxis ImageJ plugin was used to calculate velocity, directionality, and Euclidean distance from these tracks.

### Phagocytosis Assay

Phagocytic activity was assessed using the Phagocytosis Assay Kit (Cayman Chemical). Fluorescein-labeled rabbit IgG-opsonized beads were added to the primary cell culture media at a 1:200 dilution for 2 h. Cells were washed twice with assay buffer containing trypan blue to remove unbound beads and quench non-phagocytosed fluorescence. Cells were immunostained with CD11b and DAPI and imaged using immunofluorescence microscopy as described above. To assess phagocytosis, the percentage of phagocytic microglia (i.e., CD11b^+^ cells that had internalized FITC^+^ beads) was calculated. Additionally, the pixel area of fluorescent beads colocalized with CD11b^+^ microglia was measured using ImageJ and normalized to the number of cells.

### Cytokine Array

Primary microglia cells were stimulated with LPS (10 ng/mL) or DI water for 24 h beginning at 24 h after nanoparticle or PBS treatment. Cell culture supernatant was collected and centrifuged at 1500 rpm for 10 min at 4 °C to remove cells and other insoluble material. Microglial secretion of cytokines and chemokines was measured with the Proteome Profiler Rat Cytokine Array Kit (R&D Systems, ARY008) per manufacturer instructions. The supernatant (700 μL) from each group was applied to individual array membranes and incubated overnight. Twenty-nine cytokines and chemokines were spotted in duplicate and imaged simultaneously using a chemiluminescent imager. Chemiluminescent signals were detected and averaged with the Microarray Profile ImageJ plugin followed by background signal subtraction to obtain the integrated density. The nanoparticles followed by LPS treatment groups were compared to both LPS alone (positive control) and DI water (negative control) groups.

### Statistical Analysis

Sample sizes were calculated based on our previous work or power analyses. Gene expression analyses, nanoparticle characterization, and morphology were replicated to generate a sample size of *n* = 3–5/group based on prior research. For functional assays, an a priori power analysis was conducted using G*Power version 3.1.9.7 in consultation with a statistician based on preliminary data. Results indicated the suitable sample size to achieve 80% power for detecting an effect at a significance criterion of α = 0.05, was *n* = 5/group. All data are biological replicates from independent cell cultures presented as mean ± standard error of the mean (SEM) after analysis with GraphPad Prism software. As appropriate, data were analyzed using an unpaired *t* test, one-way analysis of variance (ANOVA), two-way ANOVA, χ^2^ test, or Kruskal–Wallis test. Fisher’s least significant difference test was performed as a global test to preserve the experiment-wise type I error. Post hoc analyses used Tukey’s tests or Šidák correction for multiple comparisons. A *p* value < 0.05 was considered statistically significant.

## Results

### Microglia Express Fidgetin-Like 2 (FL2) In Vivo

To confirm that FL2 is expressed in microglia in vivo, we utilized a genetically engineered mouse in which a tdTomato (tdTOM) reporter gene is inserted after the *fignl2* gene [33]. The spinal cord tissue from an adult female FL2-tdTom mouse was immunostained for tdTomato and Iba1, to label cells expressing FL2 and microglia, respectively. TdTOM signal was observed at low levels in multiple cell types, including Iba1^+^ cells, (Supplemental Fig. S1), indicating that the gene is expressed by microglia in vivo.

### Effect of Pro-Inflammatory Stimuli on FL2 mRNA Expression

**Fig. 1:**
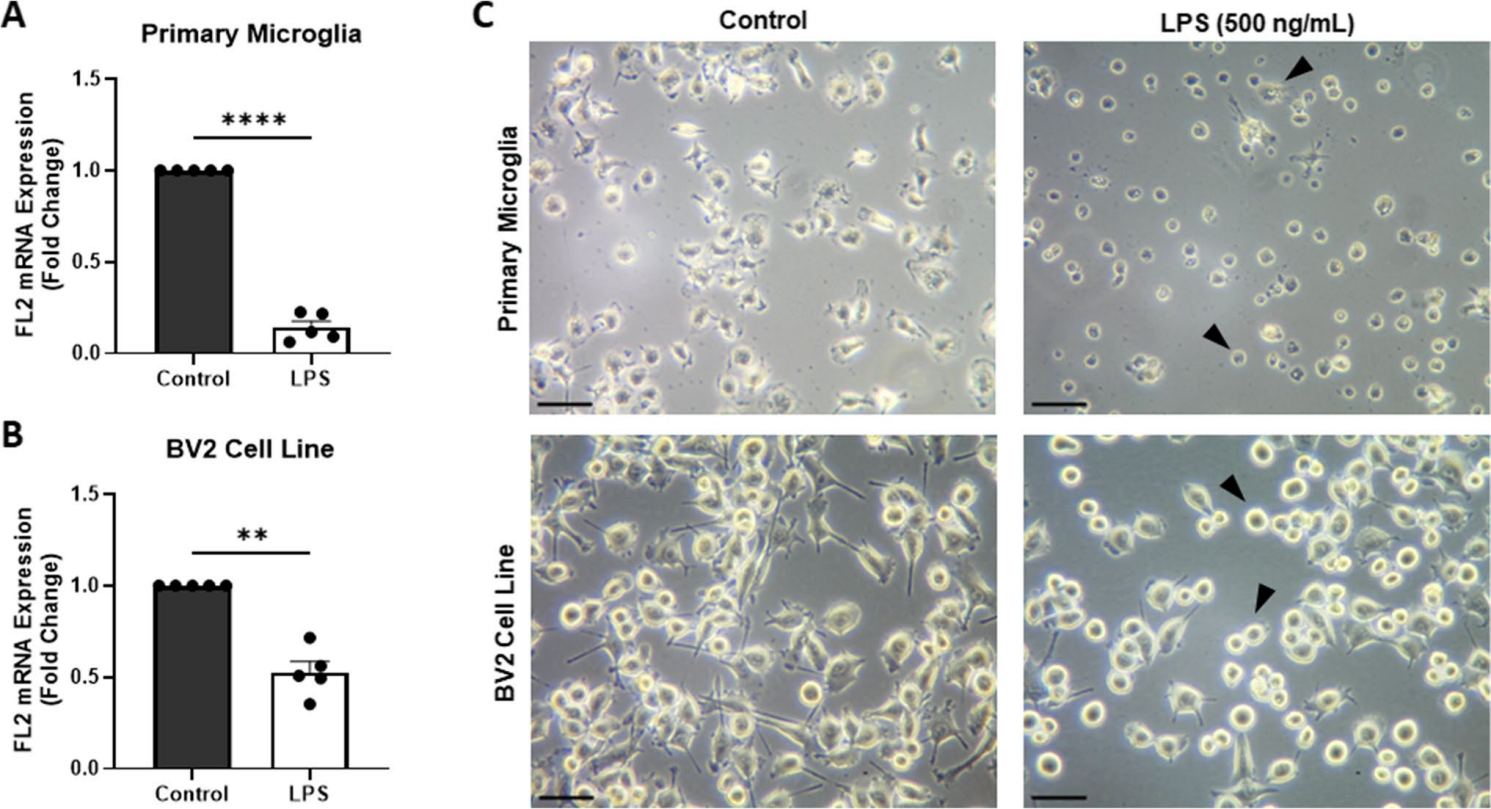
Pro-inflammatory stimuli suppress FL2 expression in microglia. RT-qPCR data for FL2 mRNA expression levels in primary rat microglia (**A**) and BV2 cells (**B**) stimulated with LPS (500 ng/mL) for 24 h. **C** Representative phase contrast images of primary microglia and BV2 morphology with water (control) or LPS (20X; scale bar = 50 µm). Arrowheads indicate activated microglia with an amoeboid morphology. Data was analyzed using Welch’s *t* tests. *n* = 5 biological replicates/group. Bars represent mean ± SEM. **p < .01, ****p < .0001

We confirmed that microglia express FL2 in vitro by RT-qPCR and investigated whether pro-inflammatory activation affects microglial FL2 expression. Both primary rat microglia and mouse BV2 microglia cells were treated with a high dose of LPS (500 ng/mL) to induce a pro-inflammatory microglial response, modeling in vivo TLR4-mediated activation. RT-qPCR was performed at 24 h to measure the relative expression of FL2 mRNA compared to control (DI water). LPS caused a significant 0.14-fold decrease in FL2 mRNA in primary rat microglia (Fig. 1A; *p* < 0.0001, Welch’s *t* test). Similarly, LPS caused a 0.52-fold decrease in FL2 mRNA in BV2 cells (Fig. 1B; *p* = 0.001, Welch’s *t* test). Both types of microglia cells exhibited morphological changes when stimulated with LPS (Fig. 1C), wherein microglia adopted a phenotype with retracted protrusions at 24 h characteristic of a more amoeboid morphology. This activation-induced morphological change has been characterized in previous studies in association with increased microtubule dynamics [14, 16, 38].

**Fig. 2:**
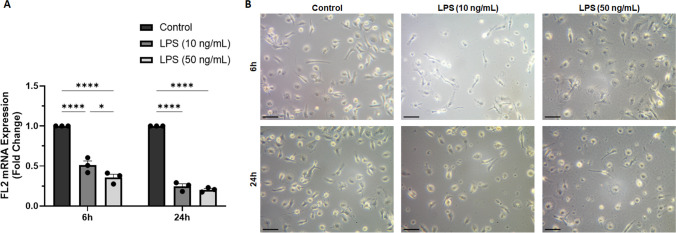
Dose- and time-dependent FL2 down-regulation in pro-inflammatory-stimulated primary microglia. Primary rat microglia cells were treated with LPS (10 or 50 ng/mL) for 6 or 24 h. **A** Gene expression as determined by RT-qPCR demonstrated that FL2 expression was significantly affected by dose and time. **B** Representative phase-contrast images of primary microglia treated with water (control) or LPS (20X; scale bar = 50 μm). Data was analyzed using a two-way ANOVA with Šidák post hoc tests. *n* = 3 biological replicates/group. Bars represent mean ± SEM. *p < .05, ****p < .0001

In subsequent experiments, we evaluated the dose- and time-dependent effects of LPS on microglial FL2 expression. Primary microglia were exposed to a low dose of LPS (10 ng/mL or 50 ng/mL) for 6 h or 24 h. FL2 expression in LPS-stimulated cells was dose- and time-dependent (Fig. 2A; Dose: *p* < 0.0001, Time: *p *< 0.001, two-way ANOVA with Šídák post hoc tests). LPS-stimulated microglia significantly downregulated FL2 expression in response to both 10 ng/mL and 50 ng/mL of LPS at 6 h (0.51-fold and 0.36-fold, respectively; *p* < 0.0001) and 24 h (0.25-fold and 0.20-fold, respectively; *p* < 0.0001) compared to control. Cells exposed to 50 ng/mL LPS expressed significantly less FL2 than cells exposed to 10 ng/mL LPS at 6 h (*p* = 0.014). While unstimulated cells exhibited a variety of morphological phenotypes with ramified processes, microglia transitioned to a mix of amoeboid and bipolar phenotypes by 6 h of low-dose LPS stimulation (Fig. 2B). Microglia significantly retracted processes by 24 h of low-dose LPS stimulation (Supplementary Fig. S2).

### Characterization of Nanoparticle-Encapsulated FL2 siRNA (SiFi2)

**Fig. 3:**
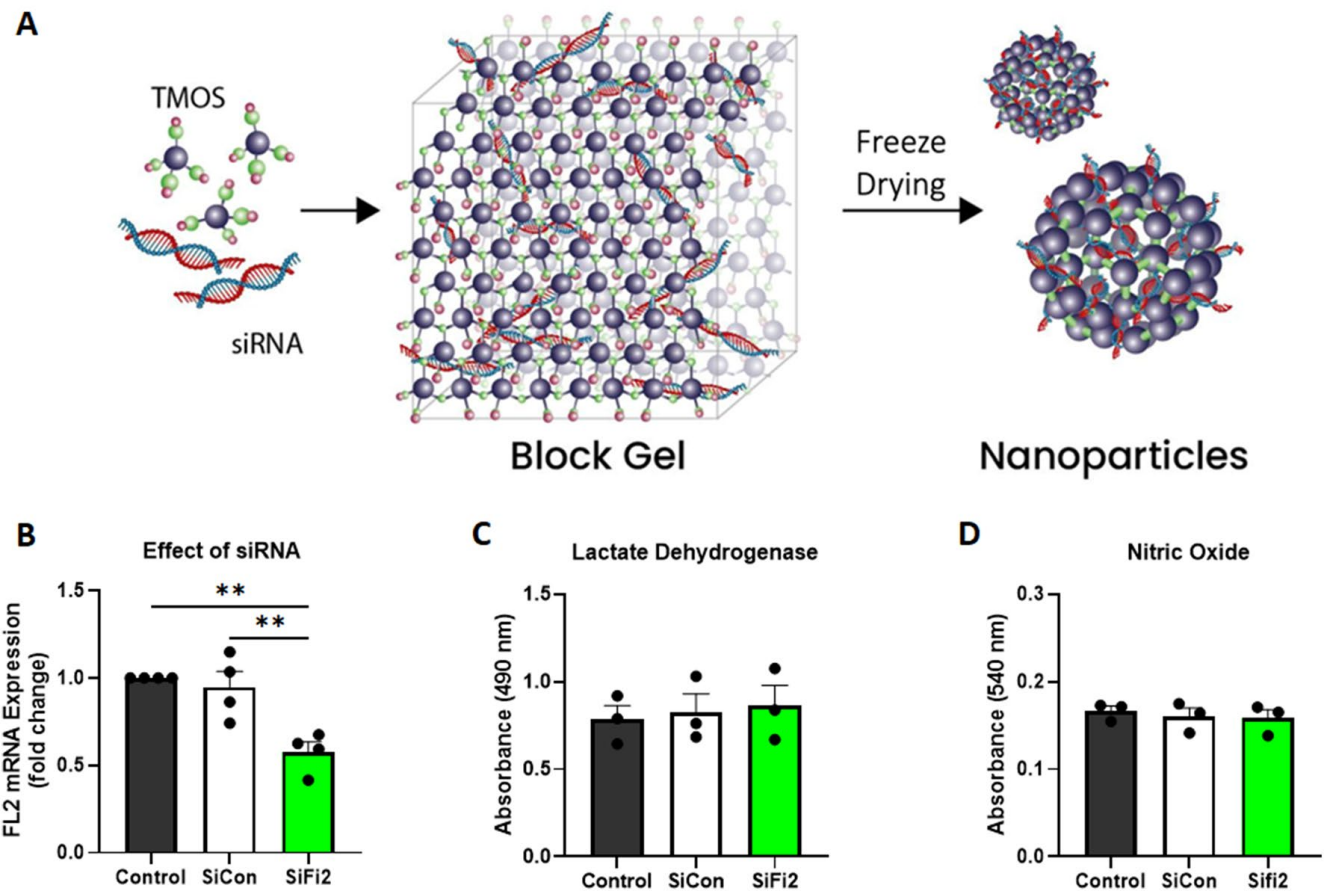
Nanoparticle-encapsulated FL2-siRNA (SiFi2) induces knockdown of FL2 in microglia. **A** Schematic of siRNAs encapsulated into silica nanoparticles. **B** RT-qPCR analysis of primary microglia treated with 20 nM SiFi2, non-targeting-siRNA (SiCon), or control (PBS) at 24 h (*n* = 4/group). LDH release (**C**), indicating cell death, and NO production (D), indicating an inflammatory response, were assayed from culture media at 24 h (*n* = 3 biological replicates/group). Data was analyzed using one-way ANOVA with Tukey’s post hoc tests. Bars represent means ± SEM. **p < 0.01

To determine if FL2 plays a role in microglial morphology and function, we sought to knockdown FL2 expression in primary microglia. We assessed whether microglial FL2 could be regulated using nanoparticle-based RNA interference. In these experiments, three FL2-targeting siRNAs were pooled and encapsulated into a TMOS nanoparticle formulation referred to as SiFi2 (Fig. 3A). This method was used by us previously to deliver siRNA in vivo as well as in cell culture [32, 33, 39]. Nanoparticles were resuspended in PBS and delivered to microglia via direct administration into the culture media. The relative levels of FL2 mRNA were determined by quantitative RT-qPCR (Fig. 3B; one-way ANOVA with Tukey’s post hoc tests). Significantly reduced FL2 levels were detected 24 h after SiFi2 treatment compared to non-targeting-siRNA nanoparticles (SiCon; *p* = 0.006) and control (PBS; *p* = 0.002). Nanoparticles did not significantly affect the percentage of CD11b^+^ microglia cells or microglia cell count (Supplementary Fig. S3). Neither SiFi2 nor SiCon caused a release of the cytotoxicity marker LDH at 24 h compared to the control group (Fig. 3C; one-way ANOVA), demonstrating that reductions in FL2 were not due to cell loss or cytotoxicity. Further, neither nanoparticle treatment induced nitric oxide (NO) production at 24 h (Fig. 3D; one-way ANOVA), demonstrating that the changes in FL2 expression were not a result of inflammatory responses to the nanoparticle treatment.

### FL2 Expression Alters Microglial Morphology

**Fig. 4:**
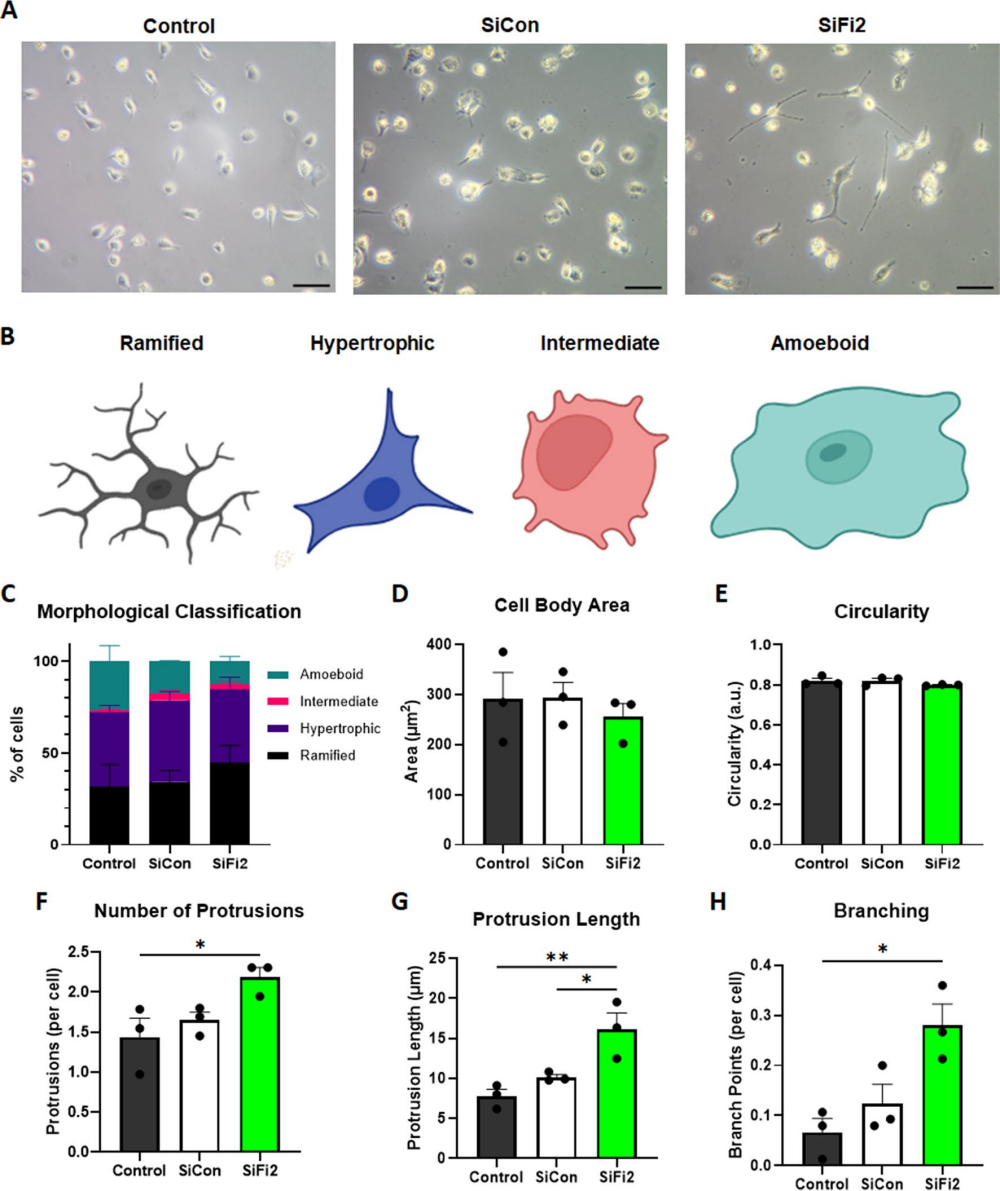
FL2 siRNA (SiFi2) alters microglial morphology. **A** Representative phase-contrast images of primary microglia cultured in the presence of control (PBS), SiCon, or SiFi2 for 24 h (20X; scale bar = 50 μm). Twenty-five cells per technical replicate (75 cells per biological replicate) were morphologically classified and quantified. **B** Diagram of microglial morphologies (ramified, hypertrophic, intermediate, and amoeboid). **C** The number of microglial morphologies was quantified as an averaged percentage of total cells. Cell body area (**D**) and circularity (**E**) were not affected by FL2 knockdown but led to significantly more cellular protrusions than controls (**F**). **G** Further, FL2 knockdown increased average protrusion length compared to SiCon and control. **H** FL2 knockdown increased the branching points of protrusions compared to control. Data were analyzed using a χ^2^ test and one-way ANOVA with Tukey’s post hoc tests. *n* = 3 biological replicates/group. Bars represent means ± SEM. *p < 0.05, **p < 0.01

We examined the extent to which FL2 knockdown changes the morphological characteristics of homeostatic microglia under phase-contrast microscopy (Fig. 4A). Cells were classified based on morphology as ramified, hypertrophic, intermediate, or amoeboid (Fig. 4B). Ramified microglia were defined as having elongated thin processes. Hypertrophic microglia were defined as having thicker, shorter processes or protrusions. Amoeboid microglia were defined as having no protrusions and an enlarged cell body. An intermediate morphology was assigned to cells between categories. Most cells in each treatment group were ramified or hypertrophic, and there were no significant differences in the percentage of cells in any category based on treatment (Fig. 4C; χ^2^ test). Quantifications were performed to assess cell bodies and cellular protrusions. Analogous to the morphological classification data, no significant differences were found in cell body area or circularity (Fig. 4D, E; one-way ANOVA). Circularity was calculated as a value from 0 to 1, 1 being a perfect circle. Upon examination of microglial membrane extensions, FL2 knockdown cells exhibited an increased average number of cellular protrusions compared to control (PBS; Fig. 4F; *p* = 0.04, one-way ANOVA with Tukey’s post hoc tests). FL2 knockdown increased the average length of protrusions compared to SiCon and control (Fig. 4G; *p* = 0.04, *p* = 0.009, one-way ANOVA with Tukey’s post hoc tests). FL2 knockdown also increased the number of protrusion branch points compared to control (Fig. 4H; *p* = 0.015, one-way ANOVA with Tukey’s post hoc tests). These results suggest that FL2 limits microtubule-based process growth and branching.

### FL2 Knockdown Increases Microglial Motility

**Fig. 5:**
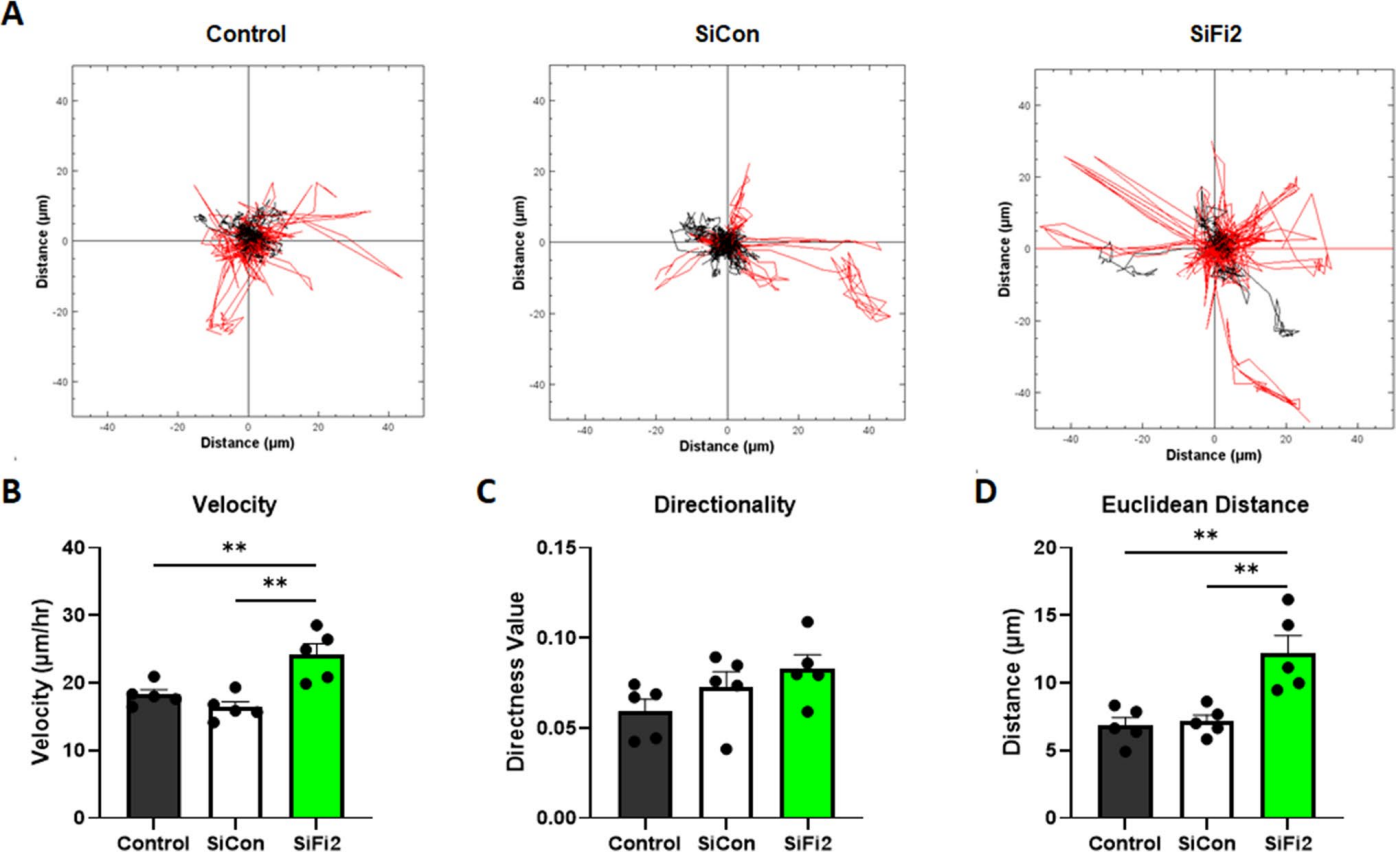
FL2-siRNA (SiFi2) increases microglial motility. **A** Representative trajectory plots from live-cell time-lapse sequences of a single ROI/group showing 30 primary cell paths. Cell soma center positions were determined every 10 min. Black and red tracks represent cells moving < 20 µm/h and > 20 µm/h, respectively, the overall mean velocity. **B** Mean velocity (accumulated distance/time) of cell motility at 24 h. **C** Quantification of persistent motility (directional movement). **D** Analysis of Euclidean distance. Data were analyzed using one-way ANOVA with Tukey’s post hoc tests. *n* = 5 biological replicates/group (120 cells/group/replicate). Bars represent means ± SEM. **p < 0.01

To examine whether FL2 expression impacts microglial migration, a basal motility assay was performed on primary microglia after FL2 was knocked down using SiFi2. Time-lapse microscopy and cell tracking were performed for 6 h beginning at 24 h post-treatment. Motility criteria excluded the cells that contacted another cell body or phagocytosed debris, to demonstrate that motility was not an effect of contact inhibition or chemical cues. In cell tracking analyses, all treatment groups displayed at least small movements back and forth, but SiFi2-treated cells demonstrated visibly larger trajectories (Fig. 5A) This migration could be observed in time-lapse videos of individual ROIs (Videos 1–3). Cells that received control (PBS), SiCon, and SiFi2 moved at an average velocity of 18.28, 16.37, and 24.13 µm/h, respectively. Statistical analysis determined that FL2 knockdown resulted in a significant increase in cell migration velocity compared to SiCon-treated cells and control cells (Fig. 5B; *p* = 0.001, *p* = 0.009, one-way ANOVA with Tukey’s post hoc tests). Directional motility was calculated as values from 0 (random) to 1 (unidirectional). Directionality values remained below 0.1 and no changes were found between groups, indicating that movement was random under all conditions (Fig. 5C; one-way ANOVA). However, the Euclidean distance (the distance between the initial and final position of the cell) was significantly higher among SiFi2-treated cells compared to SiCon-treated cells and control cells (Fig. 5D; *p* = 0.004, *p* = 0.002, one-way ANOVA with Tukey’s post hoc tests). These data indicate that FL2 regulates microglial motility.

### FL2 Knockdown Increases Microglial Phagocytosis

**Fig. 6:**
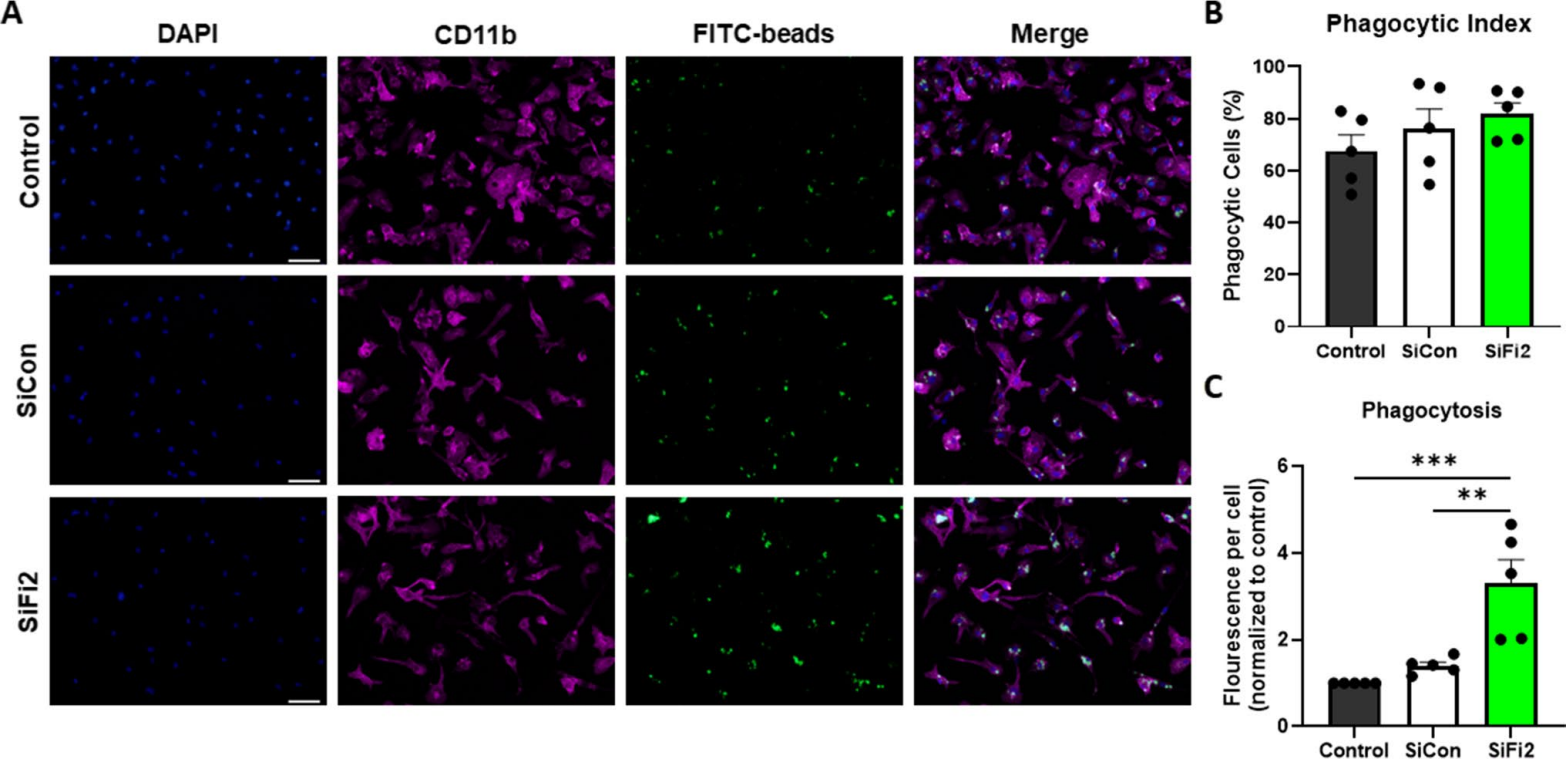
FL2-siRNA (SiFi2) increases phagocytic activity. **A** Fluorescent micrographs of primary microglia (magenta) incubated with IgG-FITC latex beads (green) for 2 h and nuclei stained with DAPI (blue) (20X; scale bar = 50 μm). **B** Quantification of the percentage of FITC^+^ cells. **C** Quantification of fluorescence colocalized within CD11b^+^ microglia normalized to the cell count with DAPI. Data were analyzed using a Kruskal–Wallis test and one-way ANOVA with Tukey’s post hoc tests. *n* = 5 biological replicates/group. Bars represent means ± SEM. **p < 0.01, ***p < 0.001

The potential for FL2 knockdown to improve phagocytic activity was evaluated in primary microglia. After 24 h incubation with control (PBS), SiCon, or SiFi2, microglia were incubated with fluorescent IgG-opsonized beads for 2 h. Cells of all treatment groups showed internalization of beads, demonstrating their capability to phagocytose (Fig. 6A). There were no significant treatment effects on the percentage of cells that were phagocytic, and a major proportion of the microglia (50%-90%) performed phagocytosis by 2 h (Fig. 6B; Kruskal–Wallis test). Quantification of internalized fluorescence density in CD11b^+^ microglia revealed that FL2 knockdown promoted a three-fold increase compared to SiCon and control groups (Fig. 6C; *p* = 0.004, *p *< 0.001, one-way ANOVA with Tukey’s post hoc tests). These findings demonstrate that FL2 is a rate-limiting factor of microglial phagocytic activity.

### FL2 Knockdown Enhances LPS-Induced Cytokine Secretion

**Fig. 7:**
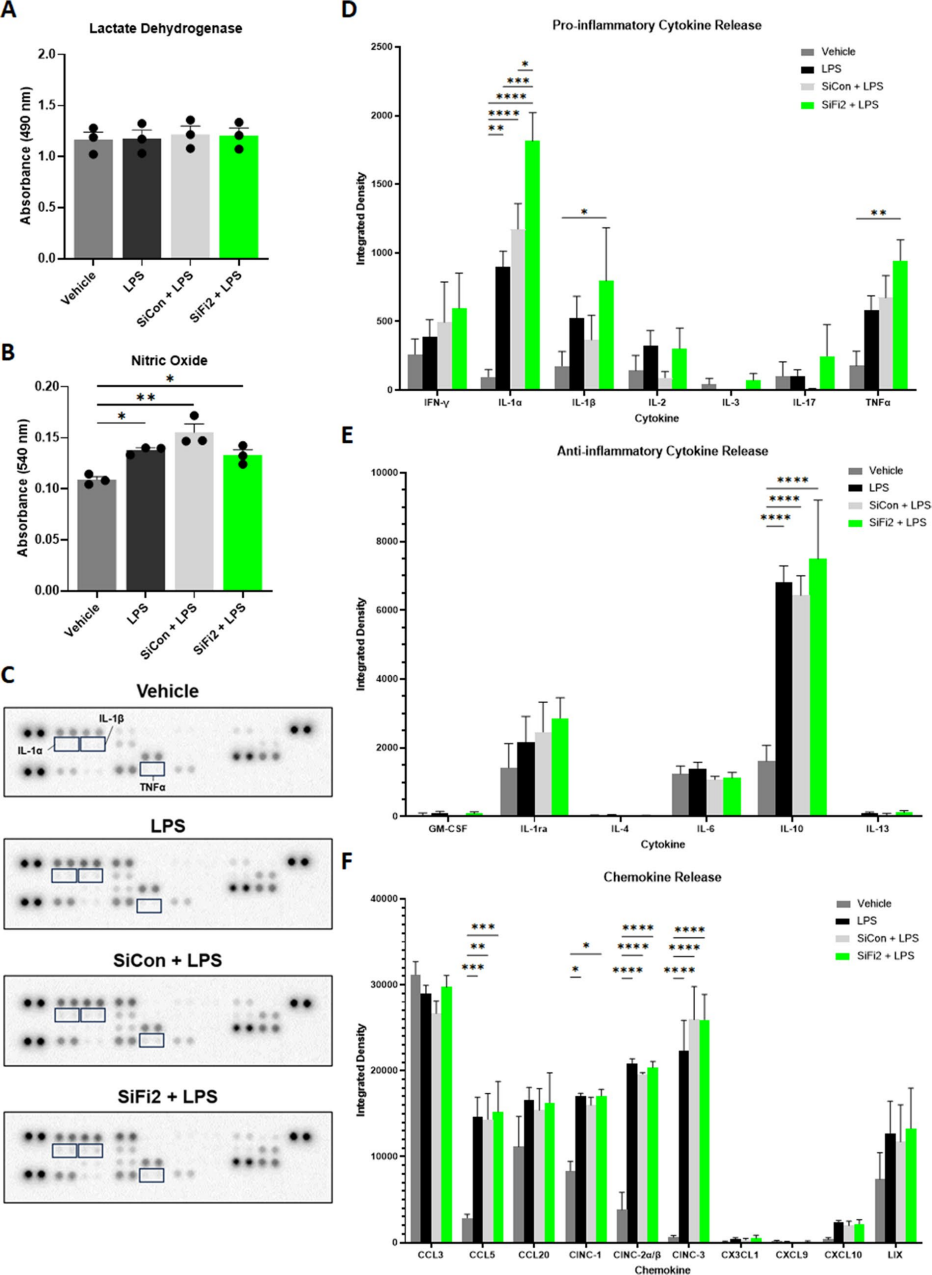
FL2-siRNA (SiFi2) enhances LPS-induced cytokine secretion. Determination of primary microglial cytotoxicity with an LDH assay (**A**) and induced NO release (**B**) after 24 h LPS exposure (one-way ANOVA). **C** Representative cytokine and chemokine array panels from cell culture supernatants after vehicle control (water), LPS alone, SiCon + LPS, or SiFi2 + LPS. Quantification of secreted pro-inflammatory cytokines (**D**), anti-inflammatory cytokines (**E**), and chemokines (**F**). Cytokine and chemokine arrays were analyzed using two-way ANOVA with Tukey’s post hoc tests. *n* = 3/group. Bars represent means ± SEM. *p < 0.05, **p < 0.01, ***p < 0.001, ****p < 0.0001

We investigated the effect of FL2 knockdown on LPS-stimulated cytokine and chemokine secretion. Primary microglia were treated with PBS, SiCon, or SiFi2 for 24 h. Then cells were exposed to low-dose LPS (10 ng/mL) or the equivalent volume of vehicle (water) for 24 h to induce an inflammatory phenotype, stimulating cytokine production. We assessed cytotoxicity and NO production as well as cytokine and chemokine release. No administered treatment caused a significant difference in LDH levels (Fig. 7A; one-way ANOVA), demonstrating no cytotoxicity and cell loss. LPS alone and with SiCon or SiFi2 significantly increased NO release compared to vehicle control (Fig. 7B; *p* = 0.020, *p* = 0.001, *p* = 0.047, one-way ANOVA with Tukey’s post hoc tests). Neither SiCon nor SiFi2 altered NO released compared to LPS alone. These observations suggest that the dose of LPS was sufficient to induce an overall pro-inflammatory response without increased cytotoxicity, and the response did not differ substantially after nanoparticle treatments.

We measured cytokine and chemokine secretion in culture supernatant using a proteome array after 24 h LPS exposure (Fig. 7C). Data were analyzed in categories determined a priori, consisting of pro-inflammatory cytokines, anti-inflammatory cytokines, and chemokines. A full diagram of the array panels and measurements of other secreted factors are in supplemental figures (Supplementary Fig. S4). Of the pro-inflammatory cytokines, LPS induced a significant increase in IL-1α secretion with LPS alone, SiCon + LPS, and SiFi2 + LPS compared to vehicle control (Fig. 7D; *p* = 0.004,* p* < 0.0001, *p* < 0.0001, two-way ANOVA with Tukey’s post hoc tests). SiFi2-induced FL2 knockdown further increased IL-1α secretion compared to LPS (*p* < 0.001) and SiCon + LPS (*p* = 0.03). SiFi2 also significantly increased the secretion of IL-1β (*p* = 0.03) and TNFα (*p* = 0.005) compared to vehicle control whereas neither LPS alone nor SiCon + LPS reached a statistically significant increase. LPS also increased secretion of the anti-inflammatory cytokine, IL-10, alone and after nanoparticle treatments compared to vehicle control (Fig. 7E; *p* < 0.0001, two-way ANOVA with Tukey’s post hoc tests). There were no statistically significant differences between LPS alone, SiCon + LPS, and SiFi2 + LPS. LPS increased secretion of the chemokines CINC-1, CINC-2α/β, CINC-3, and CCL5 compared to vehicle control (Fig. 7F; two-way ANOVA with Tukey’s post hoc tests). No differences in chemokine secretion were found between LPS, SiCon + LPS, and SiFi2 + LPS. These analyses indicate a role for FL2 in some cytokine secretion. Furthermore, the differences in cytokine or chemokine levels were neither related to altered cell viability nor an exacerbated inflammatory phenotype.

## Discussion

Our previous work identified FL2 as a novel negative regulator of cell motility and axonal growth and demonstrated that FL2 can be targeted using nanoparticle-encapsulated siRNA to promote healing in several rodent injury models, including peripheral nerve injury, cutaneous and corneal wounds [32, 33, 36]. In the present study, we identified a key role of FL2, a microtubule-severing enzyme and regulator of microtubule dynamics, in microglial structure and function. This is the first study to investigate FL2 in an immune cell, including peripheral immune cells, making these results novel. We confirmed that microglia express FL2 across multiple species and demonstrated that microglia reduce FL2 expression in response to pro-inflammatory stimuli. In primary microglia, we showed that nanoparticle-encapsulated siRNAs were a viable mechanism to knockdown FL2 expression. Morphologically, FL2 knockdown enhanced protrusion growth in homeostatic microglia. Functionally, FL2 knockdown was sufficient to increase microglial motility, phagocytic activity, and enhance pro-inflammatory cytokine secretion. These findings together suggest that FL2 contributes to microglial morphology and function.

FL2 expression was reduced in a dose- and time-dependent manner in LPS-stimulated primary rat microglia in association with the transition to a more amoeboid microglial phenotype. Reduced FL2 was replicated to a lesser extent in LPS-stimulated BV2 cells, an effect which is likely due to the high proliferation rate of this immortalized cell line reducing the effectiveness of LPS. This addresses part of the knowledge gap of how microglia regulate microtubules. First, our findings suggest that reduced FL2 expression helps facilitate the transition to a more dynamic microtubule array seen in both pro- and anti-inflammatory reactive microglia identified in prior studies [7, 14–16]. Second, these FL2 expression changes were consistent with prior literature that found FL2 severs more dynamic microtubules [32–34]. Therefore, microglia may balance FL2 expression and morphological remodeling, which is a major factor when considering the nearly constant movement of microglia or their processes and rapid phenotype changes in response to stimuli.

We successfully delivered nanoparticle-encapsulated siRNA to knock down FL2 in a somewhat heterogenous primary microglia culture system. Nanoparticle delivery had multiple advantages given the difficulty of transfecting microglia [40] and the possibility of lipid-based delivery to induce microglial activation, making lipofectamine an unfavorable siRNA delivery medium. In addition, the finding of significant group differences amongst a heterogenous culture system gives weight to the broad biological applicability of this delivery method. Although there is currently no validated antibody against rodent FL2 to evaluate protein expression, our results demonstrated that nanoparticle-encapsulated siRNA induced a 50% knockdown of FL2 mRNA at 24 h without evidence of cytotoxicity or priming NO release. This was comparable to the efficacy validated by qPCR in previous studies [32, 33], though the individual cellular uptake efficiency could not be measured. This provides further evidence that nanoparticle-encapsulated FL2 siRNA may be safe and effective for CNS treatments. A caveat to this data is that FL2 is expressed at low levels, and it is approximately 10 PCR cycles behind PGK1. However, PCR results were replicated in multiple cohorts, adding to the reliability of our results.

FL2 knockdown induced a unique microglial phenotype in which cells increased the number and length of cellular protrusions. This expands upon prior research which has demonstrated that microglial ramification and process growth are microtubule-dependent [10, 11]. Considering the findings that amoeboid LPS-stimulated microglia reduced FL2 expression, we do not conclude that reduced FL2 increases microglial ramification. Instead, FL2 knockdown microglia displayed characteristics associated with active growth and surveillance [3, 41]. Therefore, the results suggest FL2 fine-tunes directed process growth and leading-edge extensions. Future research could knock out FL2 to determine whether features of microglial morphology are dependent upon FL2 expression.

The highly motile microglial protrusions and bulbous endings are suggested to be more involved in basal motility or “surveillance” motility [41], in contrast to directed motility which can be mediated by independent stimulus-specific signal transduction pathways [42]. Based on this prior research, we investigated the effects of FL2 on basal motility and observed increased velocity and Euclidean distance upon FL2 knockdown. These results suggest that FL2 limits migration of the cell body in surveillant microglia. This finding is consistent with previous literature showing that more dynamic microtubules increase microglial motility [17, 21, 25].

While FL2 knockdown did not significantly affect the percentage of phagocytic microglial cells, it did significantly increase phagocytic activity. Therefore, FL2 may be a rate-limiting factor for microglial endocytosis. It is unclear whether FL2 knockdown in microglia increased microtubule-dependent branch-mediated phagocytosis, movement toward the beads, or both. Regardless, more efficient clearance of extracellular material would be beneficial for microglia maintaining tissue homeostasis.

We sought to test how FL2 knockdown would influence microglial cytokine secretion. Provided the evidence that inflammatory stimuli reduce FL2 expression, but wanting to induce cytokine secretion, we treated microglial with low-dose LPS for 24 h. FL2 knockdown increased IL-1α, IL-1β, and TNFα secretion. The more significant effect on IL-1α levels is likely due to its constitutive expression and secretion that precedes other cytokines upon microglial reactivity [43]. In contrast, signaling pathways control processing of other cytokines, such as NLRP3-mediated IL-1β synthesis and NF-κB-mediated TNFα production. FL2 knockdown could be directly supporting microtubule-directed vesicular transport of cytokines as well as the assembly of the NLRP3 inflammasome among other interacting signaling pathways. These findings suggest that earlier reductions of FL2 expression support more rapid cytokine secretion to begin a positive feedback loop, amplifying inflammatory signals.

It has been documented that changes in FL2 expression can directly affect microtubules, and indirectly affect intracellular pathways, such as RhoA-mediated focal adhesion turnover [34]. Thus, the observed morphological and functional changes in microglia could be attributed to multiple intracellular mechanisms underlying microglial dynamics.

## Conclusion

Our investigation into the role of a specific microtubule-severing enzyme on microglial morphology and function reveals that FL2 may be a crucial regulator for both maintaining ramified homeostasis and rapid microglial responses. Our previous in vitro work has provided evidence that FL2 is a potential therapeutic target for promoting axonal regeneration and nervous system repair [33]. We now show that FL2 is a viable target for altering microglial responses as well and suggest that downregulating FL2 may work to increase or accelerate central as well as peripheral immune reactions to various stimuli, as a therapeutic consideration for future work. Our findings indicate that FL2 knockdown and increased microtubule dynamics allow microglia to function more efficiently. Future work will determine whether FL2-based therapies improve recovery and regeneration after CNS injury and investigate how such therapies may affect microglial responses in vivo.

## Supplementary Information

Below is the link to the electronic supplementary material.

Supplementary Methods (DOCX 34 kb)

Supplementary Fig. S1 (PNG 571 kb)

High resolution image (TIF 3101 kb)

Supplementary Fig. S2 (PNG 30 kb)

High resolution image (TIF 993 kb)

Supplementary Fig. S3 (PNG 245 kb)

High resolution image (TIF 2178 kb)

Supplementary Fig. S4 (PNG 34 kb)

High resolution image (TIF 2809 kb)

Video 1 (AVI 2033 kb)

Video 2 (AVI 1821 kb)

Video 3 (AVI 1947 kb)

## Data Availability

The datasets generated and/or analysed during the current study are available in the OSF repository, 10.17605/OSF.IO/N78BQ

## Abbreviations

CNS: Central nervous system
FL2: Fidgetin-like 2
LDH: Lactate dehydrogenase
LPS: Lipopolysaccharide
NO: Nitric oxide
SiCon: Control siRNA nanoparticles
SiFi2: FL2 siRNA nanoparticles
TMOS: Tetramethyl orthosilicate
