# Supplementary material for: Downregulation of Fidgetin-Like 2 Increases Microglial Function: The Relationship Between Microtubules, Morphology, and Activity
Source: Mol Neurobiol. 2024 Aug 19;62(3):2726–39. doi: 10.1007/s12035-024-04404-0 (PMC11790376; doi:10.1007/s12035-024-04404-0)
Supplement: Supplementary file 1 — (DOCX 34 kb) [file 12035_2024_4404_MOESM1_ESM.docx]

**Supplementary Methods**

**Immunohistochemistry of FL2-tdTOM mouse spinal cord**

We utilized a genetically engineered mouse (GenOway), in which a tdTomato reporter gene was inserted after the *fignl2* gene (33), to examine whether FL2 is expressed in microglia in the spinal cord *in vivo*. An internal ribosome entry sequence was placed between the fignl2 and tdTOM so that the protein products are expressed separately.

To collect spinal cord tissue, mice were anesthetized with Euthasol and intracardially perfused with saline followed by 10% buffered formalin. The spinal cord was dissected and post-fixed for 24 hours in 10% buffered formalin and cryoprotected in 30% sucrose in PBS solution for several days before embedding in O.C.T. The tissue was cryosectioned longitudinally in 10 μm sections. Sections were stained for tdTOM and Iba1 using a rabbit anti-RFP antibody (Rockland, 600-401-379) and a mouse anti-Iba1 antibody (Abcam, ab283319). Sections were blocked in blocking buffer (10% donkey serum, 1% BSA, 0.1% triton X in PBS) for one hour at room temperature followed by one hour blocking with M.O.M. blocking reagent (Vector Biolabs), prior to adding primaries for overnight incubation at 4 C. Primaries were diluted 1:200. After washing with PBS samples were incubated with Alexa Flour 647 goat anti-rabbit (Invitrogen, A32733) and Cy2 Donkey anti-mouse (Jackson Immunoresearch, 715-225-150) at dilutions of 1:200 in blocking buffer for 1 hour at room temperature, stained with DAPI (Life Technologies, Ref. D1306) in PBS and mounted. Slides were imaged using an EVOS Fluorescent Auto (Life Technologies) at fixed light and exposure settings and channels merged in ImageJ.

**Supplementary Figure S1** **Fidgetin-like 2 is expressed in microglia in the adult mouse spinal cord.** (A) Immunomicrographs of a longitudinal spinal cord section from a young adult FL2-tdTomato female mouse immunostained for tdTomato (red) and Iba1 (green) to mark cells expressing FL2 and microglia, respectively. Arrowheads indicate FL2 and Iba1 co-localization. (B) Micrographs of control-stained tissue from a similar area of the spinal cord, for which only secondary antibodies and no primary antibodies were used. (20X; scale bar = 100 µm).

**Supplementary Figure S2 Microglial morphological changes after low-dose LPS.** Primary rat microglia were treated with 10 ng/mL LPS or water (control) for 6 h or 24 h. Quantification of microglial morphology revealed that LPS reduced the average protrusion length characteristic of retracted processes at 24 h. There were no statistically significant differences in the average number of protrusions (B) or branch points (C) per cell. Data was analyzed using two-way ANOVA with Šidák *post hoc* tests. n = 3 biological replicates/group. Bars represent mean ± SEM. **p < 0.01

**Supplementary Figure S3 Primary rat microglia culture purity and proliferation.** (A) Representative images of cell cultures immunostained for DAPI (blue) and CD11b (red) (20X; scale bar = 50 µm). The asterisk indicates a CD11b negative cell. (B) Percentage of CD11b^+^ cells after control, SiCon, or SiFi2 treatment. Data was analyzed using a Kruskal-Wallis test. (C) Quantification of CD11b^+^ cell counts normalized to control. Data was analyzed using one-way ANOVA. n = 5 biological replicates/group. Bars represent mean ± SEM.

**Video 1** Representative video of control-treated primary microglia under live-cell time-lapse imaging (6 h at 3 frames per second).

**Video 2** Representative video of SiCon-treated primary microglia under live-cell time-lapse imaging (6 h at 3 frames per second).

**Video 3** Representative video of SiFi2-treated primary microglia under live-cell time-lapse imaging (6 h at 3 frames per second).

**Supplementary Figure S4** **Proteome Profiler.** (A) Array spot coordinates. Each duplicate is labeled as the respective cytokine, chemokine, RS (Reference Spot), or NC (Negative Control). (B) Quantification of secreted proteins that were not considered for grouped analyses. Data were analyzed using two-way ANOVA with Tukey’s *post hoc* tests. n = 3 biological replicates/group. Bars represent means ± SEM.
