# Supplementary figures and images for: Downregulation of Fidgetin-Like 2 Increases Microglial Function: The Relationship Between Microtubules, Morphology, and Activity
Source: Mol Neurobiol. 2024 Aug 19;62(3):2726–39. doi: 10.1007/s12035-024-04404-0 (PMC11790376; doi:10.1007/s12035-024-04404-0)

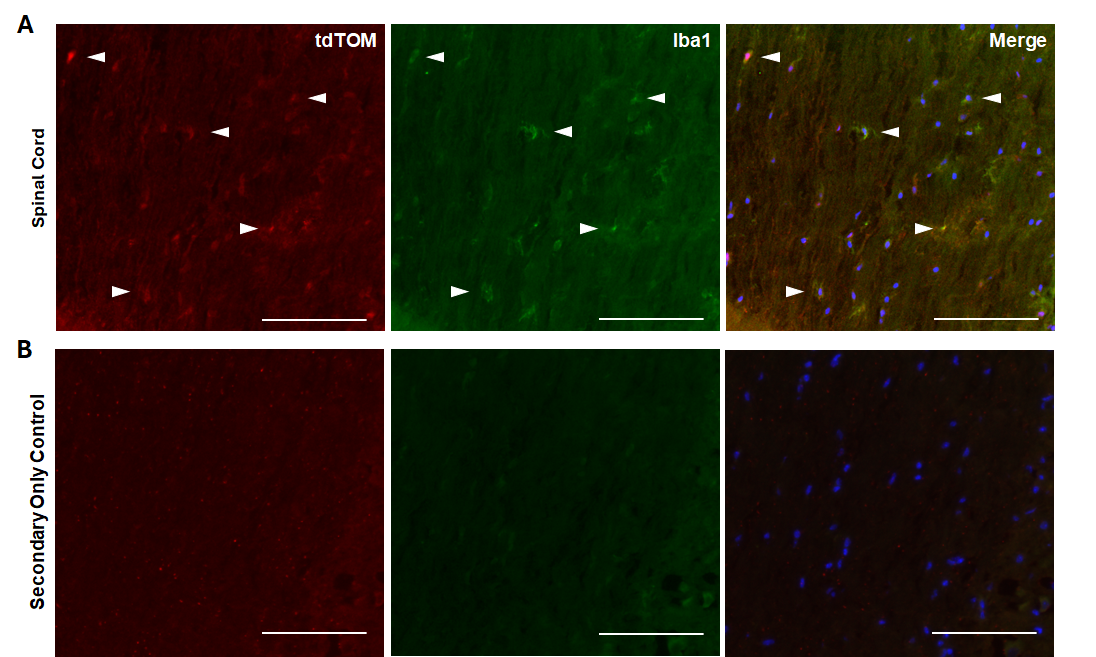

Supplement: Supplementary file 2 — (PNG 571 kb) [file 12035_2024_4404_Fig8_ESM.png]

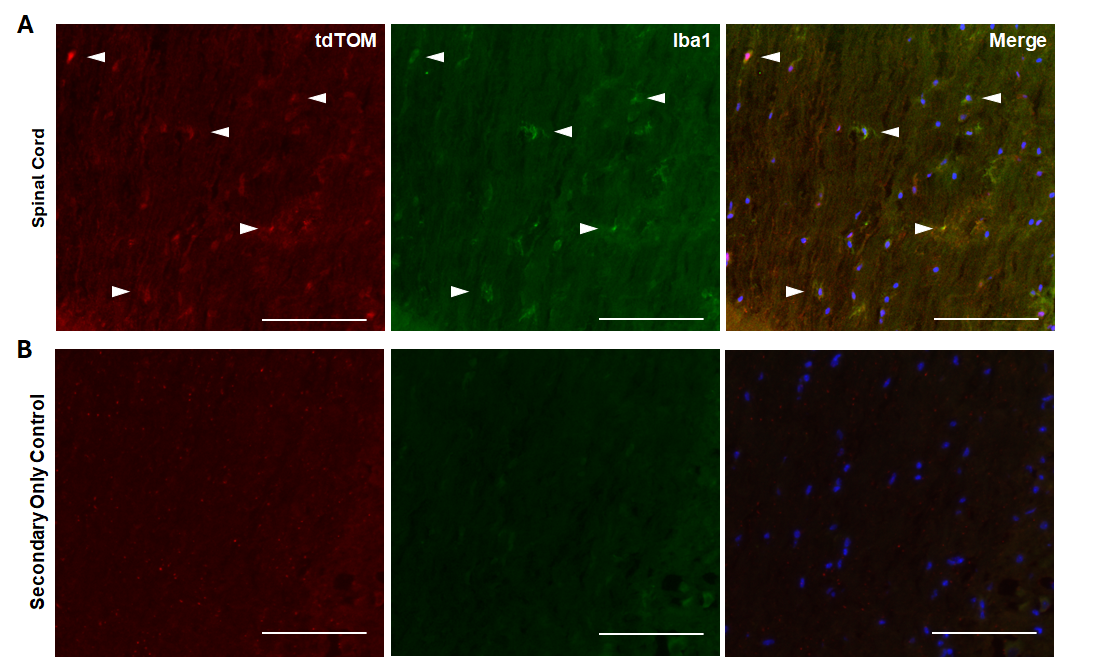

Supplement: Supplementary file 3 — High resolution image (TIF 3101 kb) [file 12035_2024_4404_MOESM2_ESM.tif]

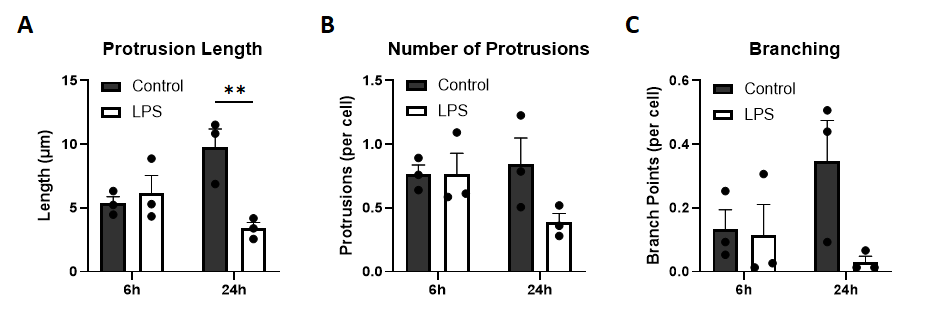

Supplement: Supplementary file 4 — (PNG 30 kb) [file 12035_2024_4404_Fig9_ESM.png]

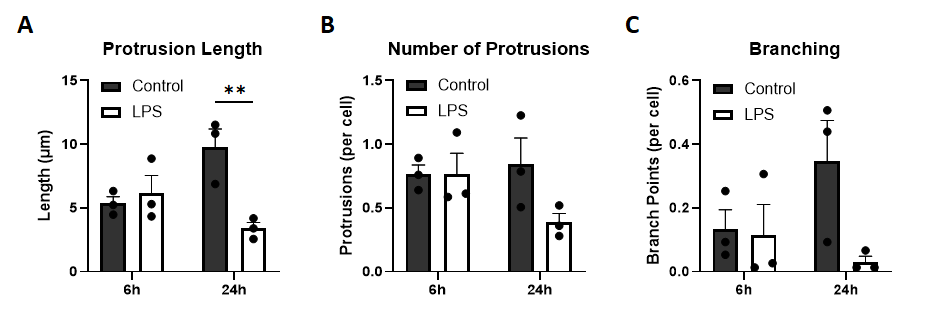

Supplement: Supplementary file 5 — High resolution image (TIF 993 kb) [file 12035_2024_4404_MOESM3_ESM.tif]

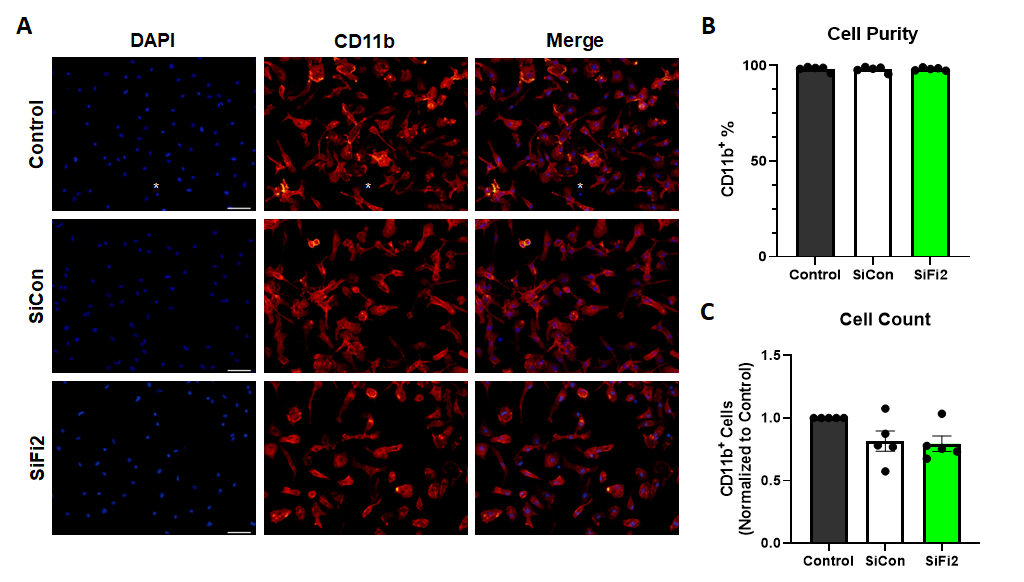

Supplement: Supplementary file 6 — (PNG 245 kb) [file 12035_2024_4404_Fig10_ESM.png]

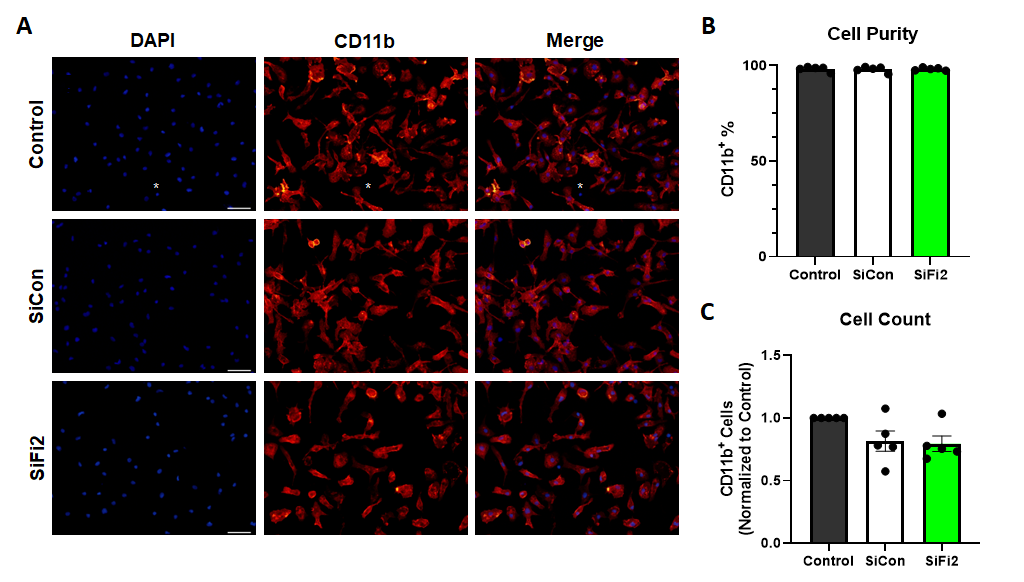

Supplement: Supplementary file 7 — High resolution image (TIF 2178 kb) [file 12035_2024_4404_MOESM4_ESM.tif]

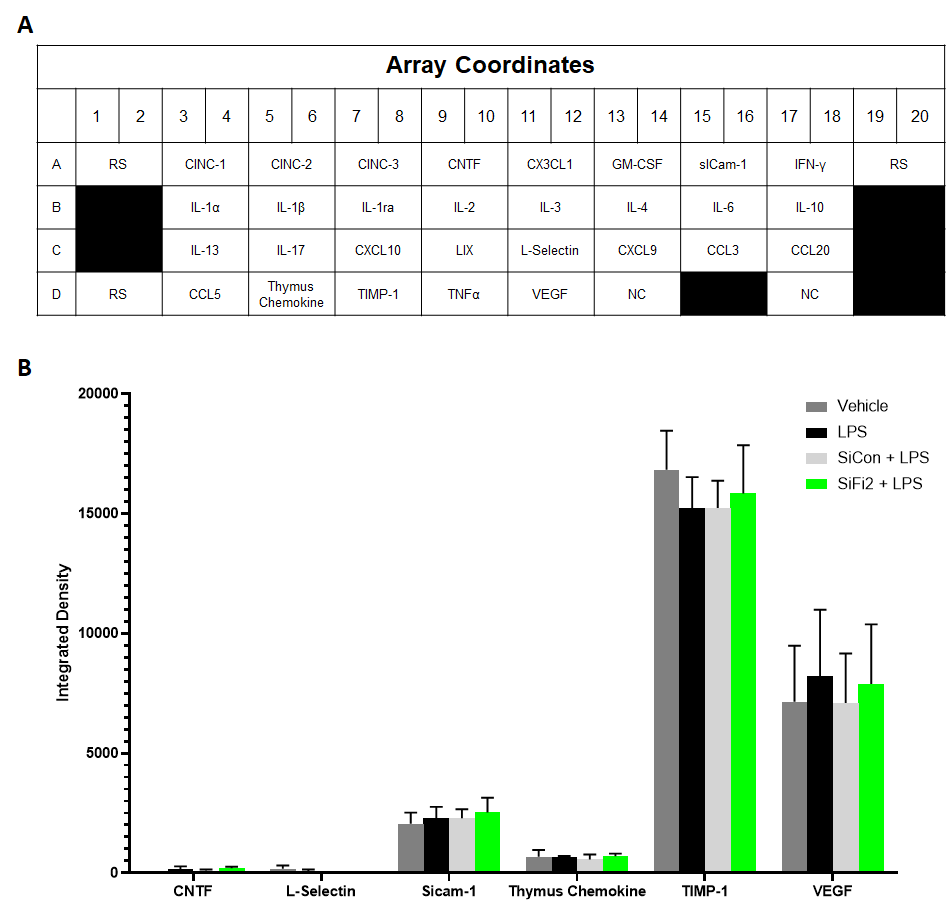

Supplement: Supplementary file 8 — (PNG 34 kb) [file 12035_2024_4404_Fig11_ESM.png]

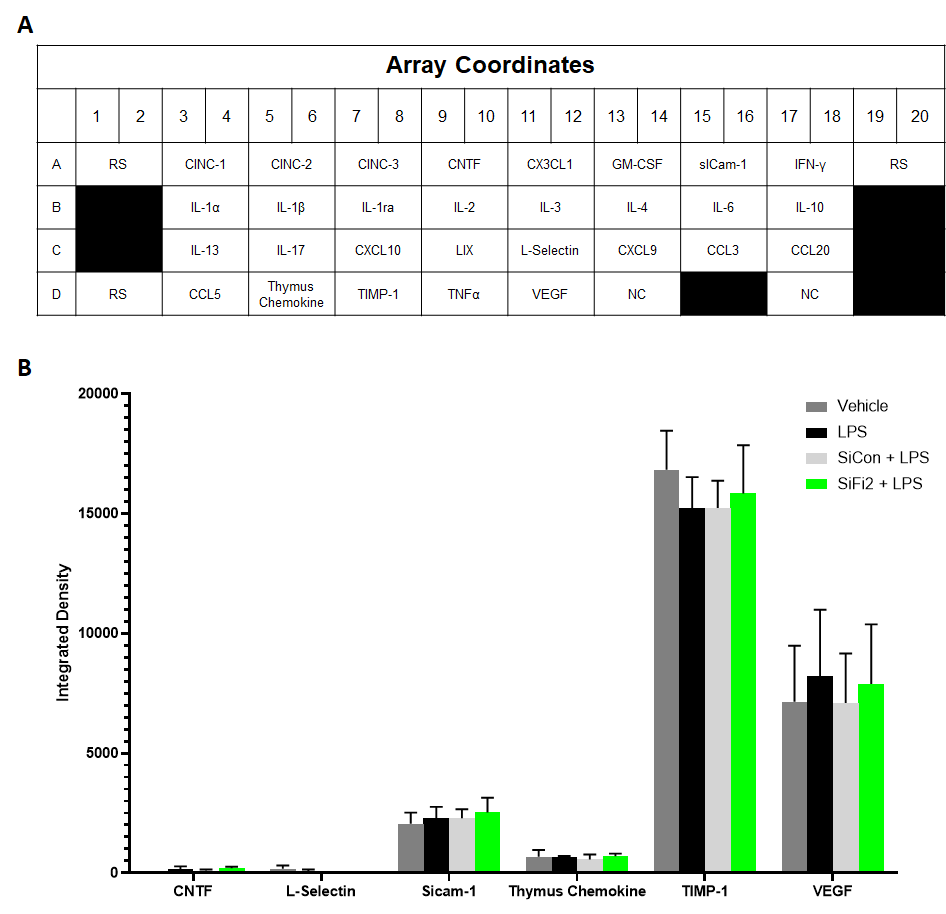

Supplement: Supplementary file 9 — High resolution image (TIF 2809 kb) [file 12035_2024_4404_MOESM5_ESM.tif]
